# Hall effect FEM modeling using FreeFEM++: Illustration on a cross-shaped graphene sensor

**DOI:** 10.1371/journal.pone.0357905

**Published:** 2026-09-24

**Authors:** Vigneswaran Ravi, Sophias Boko, Christophe Coillot, Sébastien Nanot

**Affiliations:** Laboratoire Charles Coulomb (L2C), CNRS, Université de Montpellier, Montpellier, France; UFCSPA: Universidade Federal de Ciencias da Saude de Porto Alegre, BRAZIL

## Abstract

The Hall effect, which occurs in metals and semiconductors, is used in numerous applications, particularly in the fabrication of magnetic field sensors. This phenomenon is influenced by the physical properties of the material (electron mobility, doping), the geometry of the device, and the size and position of the metallic contacts. The analytical model has limitations in accurately capturing all these phenomena. Therefore, in this work, we propose a finite element model using FreeFEM++ to accurately model the Hall effect. The validity of this model is assessed by comparing it to analytical ones. The effect of physical defects on the electrical measurements, which cannot be analytically calculated, is explored to illustrate the usefulness of this model under realistic conditions. These simulations can be used both as teaching tools, to illustrate this physical effect and as research tools, as demonstrated in the case of a graphene-based transistor. The reported simulation script is openly shared.

## 1 Introduction

The Hall effect was discovered in conducting materials almost 150 years ago [1]. Since then, it has found widespread application in science and technology, ranging from material characterization to everyday applications, and even lead to the discovery of its quantum counterpart [2]. Hall sensors utilize the Hall effect to detect the magnetic field by measuring the Hall voltage. Such sensors find applications in various areas [3], including compasses, motor positioning, contactless current sensing or power measurement, and non-destructive measurement. However, there are still challenges to improve their magnetic field resolution, reduce their size and their power consumption. For these reasons, graphene, a 2D material composed of a single layer of carbon atoms arranged in a hexagonal lattice structure [4], has rapidly been considered a promising material for such sensing applications. Its scalability, stability and reproducibility are currently extensively studied to assess its potential for actual applications [5–8]. So far, its magnetic field detection limit has been demonstrated to reach almost $700\;\;nT\;Hz^{-1/2}$ [9].

In fact, the Hall effect measurements are not just restricted to magnetic field sensing. When combined with resistivity measurements, they provide insight into material propertie, such as carrier concentration and mobility. Resistivity can be obtained by measuring non-local voltages using the so-called van der Pauw (vdP) method, which has the advantage of being performed using the same four contacts as the Hall measurements. These two characterization methods (Hall and vdP) are widely used four probe techniques [10] applied to thin material samples. However, various practical considerations and parameters limit their applicability for material characterization, including anisotropic conductivity, sample shape, size and location of the metallic contacts, and the presence of defects.

For isotropic and homogeneous materials, a simple analytic model can be used to determine the sensitivity of a sensor and to relate the charge carrier density and mobility with the measured parameters. When a current, *I*, flows through a conducting plane subject to a transverse magnetic field, *B*, a Hall voltage, $V_{H}$, normal to both *I* and *B* appears. This effect forms the basis of Hall sensors. The Hall voltage can be derived from the Lorentz force as $V_{H}=K_{H}IB/t$, where $K_{H}$ is the Hall coefficient, and *t* is the thickness of the film (in m). Under the assumption of a one-carrier regime, the Hall coefficient (in Ω/T) is given by:


$$\begin{array}{c} K_{H}=\frac{1}{nq} \end{array}$$ (1)

where *n* is the charge carrier density (in $m^{-3}$) and *q* = +*e* for holes or q=−e for electrons and *e* is the elementary charge. The sensor sensitivity becomes $S=|K_{H}|$ (in *V*/(*A*.*T*)). In 2D materials constituted with a few atomic layers, like graphene, (or in any bidimensional electron gas like in quantum wells), the thickness vanishes and the Hall voltage is expressed as:


$$\begin{array}{c} V_{H,2D}=K_{H}IB=\frac{IB}{n_{2D}q} \end{array}$$ (2)

where *n*_2*D*_ is the 2D carrier concentration (in *m*^-2^). It should be noticed that near charge neutrality both electrons and holes contribute simultaneously to charge transport in graphene. This cannot be described by a simple one-carrier Drude model. In the following, only hole charge carriers are considered, corresponding to the most typical intrinsic doping of graphene.

For a complete material characterization, Hall measurements are combined with longitudinal resistivity measurements, $\rho_{xx}=\rho$. This combination gives access to the carrier mobility, μ through:


$$\begin{array}{c} \rho^{-1}=n|q|\mu =\mu /K_{H} \end{array}$$ (3)

The resistivity can be obtained using the same electrodes employing the van der Pauw (vdP) method [11]. It consists of a four-probe non-local measurement in which the contacts are placed along the perimeter of the sample. The current is injected through two neighboring contacts and the voltage, $V_{vdP1}$, is measured between the remaining two contacts. The probe configuration is then rotated by 90° and the voltage, $V_{vdP2}$, is measured. Dividing each voltage by the injected current yields two resistances, $R_{vdP1}$ and $R_{vdP2}$. From these resistance values, the resistivity of a 2D material can be obtained by solving the following equation:


$$\begin{array}{c} e^{-\frac{\pi R_{vdP1}}{\rho_{2D}}}+e^{-\frac{\pi R_{vdP2}}{\rho_{2D}}}=1 \end{array}$$ (4)

In cases where the measured resistances are close to each other, Eq. (4) can be approximated by:


$$\begin{array}{c} \rho_{2D}=\frac{\pi }{ln(2)}R_{vdP} \end{array}$$ (5)

where $R_{vdP}$ is the averaged vdP resistance, $R_{vdP}=(R_{vdP1}+R_{vdP2})/2$. For improved accuracy, the measurements can be averaged over two additional probe configuration rotations, and reversing the current direction for each of these configurations (up to 8 measurements).

In the case of materials with anisotropic conductivity, some studies proposed improvements to the vdP method [10]. To deal with isolated defects, studies suggest interpreting the vdP equation as an inequality in the presence of a hole. This approach makes it possible to determine the resistivity of a material containing a single isolated hole [12] or even multiple holes [13] from vdP measurements. In these studies, some aspects, such as anisotropic materials and isolated holes, are addressed using analytical equations. Although these models are elegant, they are limited to specific cases and geometrical configurations.

For this purpose, the Finite Element Method (FEM) provides a powerful numerical approach for modeling realistic devices by solving the governing differential equations over a discretized finite element space. To the best of our knowledge, only a few studies have used a FEM software to model and investigate the Hall effect in semiconductors to get deeper insights on geometrical effects, design optimization, and metallic contact effects on the sensitivity [14–16]. An advanced modeling of graphene Hall transistors has also been proposed [17]. In contrast, free and open-source software programs provide an attractive alternative for solving partial differential equations (PDEs), facilitating the sharing and reproducibility of computational models for research and educational purposes. They make it easier to share the developed models for research and educational purposes. Among the free alternatives, FreeFEM [18] offers a powerful possibility with a wide user-community. FreeFEM was successfully used in Ref. [14] to investigate thermal drift in gated GaAs Hall effect sensor.

In the present work, we extend this study in various directions: i) physical description of the problem closer to experimental conditions (boundary conditions and metallic contacts), ii) the combined model of the Hall and vdP resistances, iii) investigation of the effect of isolated holes on the electrical properties, iv) a detailed description of the FreeFEM model. The FreeFEM simulation script is made accessible, and we illustrate our simulation script on a cross-shaped Hall graphene transistor assuming a one carrier model Eq (2).

## 2 FEM methodology

### 2.1 Generalities

The FEM method consists of discretizing the problem domain into a number of connected subdomains, usually triangular, known as **finite elements**. Together, these elements form the **mesh**. The physical problem must be described by a single differential equation for a given physical quantity, *u*. Following this, the equation is expressed in its weak form, or a variational form of the equation, in which the equation is multiplied by a test vector and integrated over the surface under consideration. Finally, the appropriate global boundary conditions are written to coincide with realistic conditions. The weak form of the equation is then locally solved at each point on the mesh, while satisfying the boundary conditions (Galerkin method). More details on the fundamentals of the finite element method can be found in various books and sources, such as [19].

**Fig 1:**
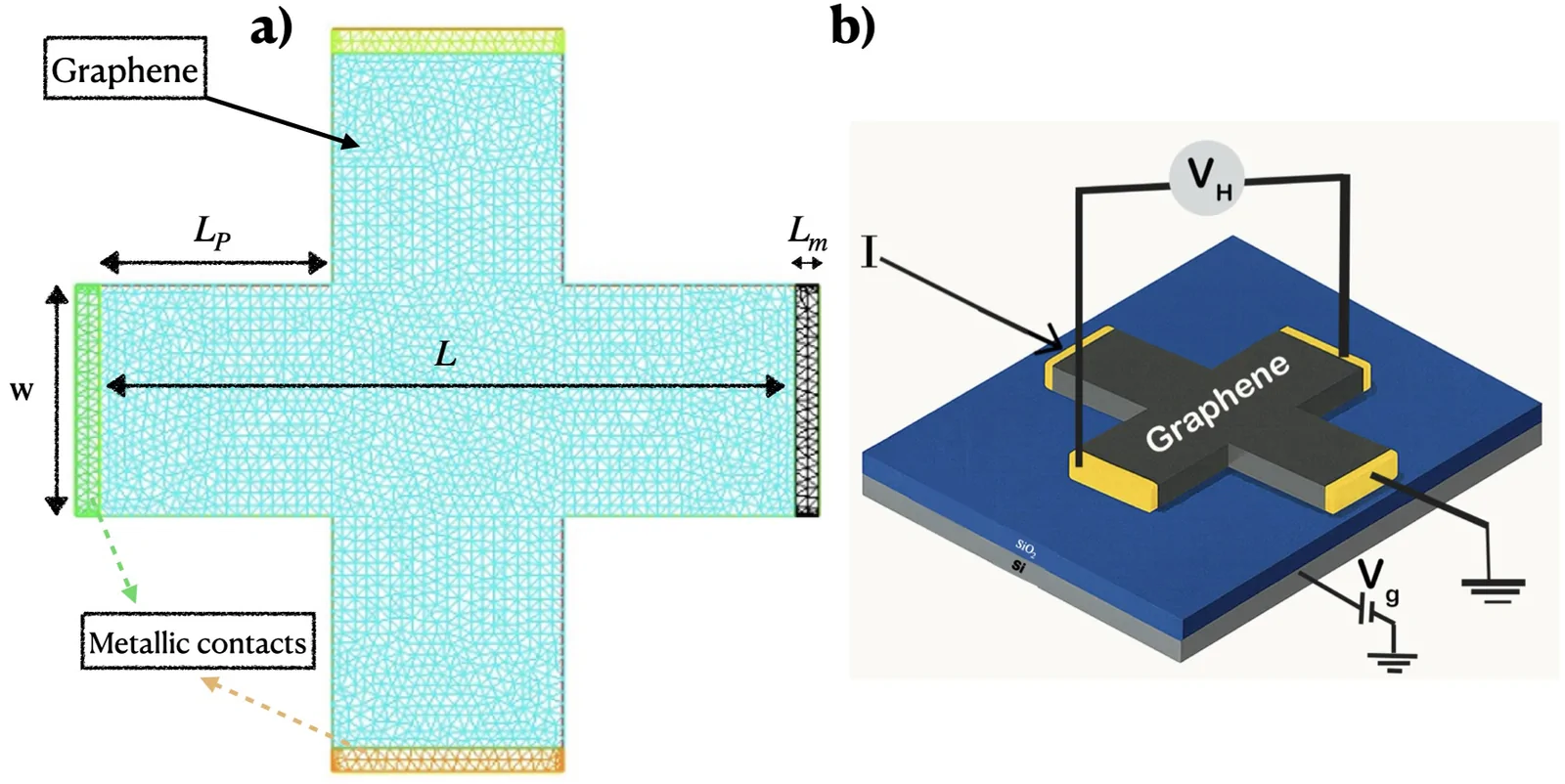
Graphene FET Hall cross: Mesh and device. (a) Illustration of the meshed cross-shaped geometry of length *L* and width *W* with metallic contacts of length $L_{m}$. (b) Graphene transistor configuration indicating the parameters such as bias current (*I*), Hall voltage ($V_{H}$) and gate voltage ($V_{g}$) which modulates the carrier concentration.

In this section, we define the Hall sensor as a cross-shaped conducting 2D material with 4 metallic contacts and a tunable carrier density through a backgate (see Fig 1). The equation for the local electrostatic potential *u* = *u*(*x*,*y*) accounts for a current flowing from left to right under a magnetic field applied perpendicular to the plane, while the Hall voltage is measured between the top and bottom contacts.

### 2.2 Geometry description of a cross-shaped Hall sensor.

The cross-shaped geometry of the Hall sensor consists of four intersecting branches of a conducting 2D material with metal contacts (gold) located at the end of each branch (the magnetic field *B* is out-of-plane) (cf. Fig 1.b). The aspect ratio of the Hall cross is defined as *L*/*W*, where *L* is the length of the sensor and *W* is the branch width. This ratio has practical implications on the sensitivity, the voltage offset and the intrinsic noise of Hall sensors. he mesh is selected as a compromise between the level of accuracy (resolution) and the computational time, as discussed in more detail in the Results section.

## 3 Physical formalism of the Hall effect

To simulate the Hall effect in graphene, a one-carrier model is assumed, allowing the reliability of the simulation results to be validated by comparison with the analytical model. First, a differential equation describing the physical phenomenon is derived. The only physical variable is the electrical potential (*u*) which is a function of the position (*x*,*y*) within the Hall cross, and therefore, over the computational mesh. The differential equation also depends on various parameters: the magnetic field, *B*, the injected current *I* and the charge carrier density, *n*(*x*,*y*). This physical equation is then further simplified to obtain the variational (or weak) form. Finally, the appropriate boundary conditions are stated to properly describe the physical system.

### 3.1 Physical description of the Hall effect

The partial differential equation (PDE) describing the effect of a magnetic field on the local potential, *u* = *u*(*x*,*y*), in a graphene transistor is deduced from a combination of the Lorentz force and the charge continuity equation (derivation of the PDE is detailed in S1 Appendix). The PDE of the Hall effect is given by:


$$\begin{array}{c} \frac{\mu C_{g}}{\left(1+(\mu B_{z}{)}^{2}\right)}[((u-V_{g})+\frac{en^{*}}{C_{g}})\nabla^{2}u+(\frac{\partial u}{\partial x}{)}^{2}+(\frac{\partial u}{\partial y}{)}^{2}]=0 \end{array}$$ (6)

where $B_{z}$ (in *T*) is the transverse component of the magnetic field, and μ (=μ(x,y)) is the charge carrier mobility of the material (μ depends on the region: $\mu =\mu_{g}$ in the graphene and $\mu =\mu_{m}$ in the metal). The effective carrier density, *n*, is modulated by a backgate voltage, $V_{g}$, through a planar capacitive coupling:


$$\begin{array}{c} n(x,y)=\frac{C_{g}}{e}(u-V_{g})+n^{*} \end{array}$$ (7)

where $C_{g}$ is the gate capacitance, $n^{*}$ is the charge carrier density at $V_{g}=0V$, corresponding to the residual doping.

### 3.2 Variational form

The derivation of the weak form of Eq (6) allows us to solve a linear integral problem in *u*(*x*,*y*) using the FreeFEM++ software with appropriate boundary conditions (N.B. the effect of non-linear terms in *u*(*x*,*y*) are neglected, as discussed in S1 Appendix):


$$\begin{array}{cc} & \iint_{\begin{array}{c} S \end{array}}\frac{-\mu C_{g}}{1+(\mu B_{z}{)}^{2}}\left(\frac{en^{*}}{C_{g}}-V_{g}\right)\nabla u\nabla vdS\\ & \;\;-\int_{\begin{array}{c} \partial S \end{array}}\frac{-\mu C_{g}}{1+(\mu B_{z}{)}^{2}}\left(\frac{en^{*}}{C_{g}}-V_{g}\right)\nabla u\cdot \hat{n}\;v\;dl=0 \end{array}$$ (8)

where *v* is the test vector, $\hat{n}$ is the unit vector normal to the boundary, *dl* is the infinitesimal line element along the boundary (∂S) and *dS* is the infinitesimal surface element over the surface (*S*). The first term of the weak formulation corresponds to the variational form of the Laplace operator. The second integral term represents the boundary condition of the problem, which naturally originates from the derivation of the weak form.

### 3.3 Boundary conditions

Proper boundary conditions are crucial for modeling the intended problem as close as possible to realistic device conditions.

**Fig 2:**
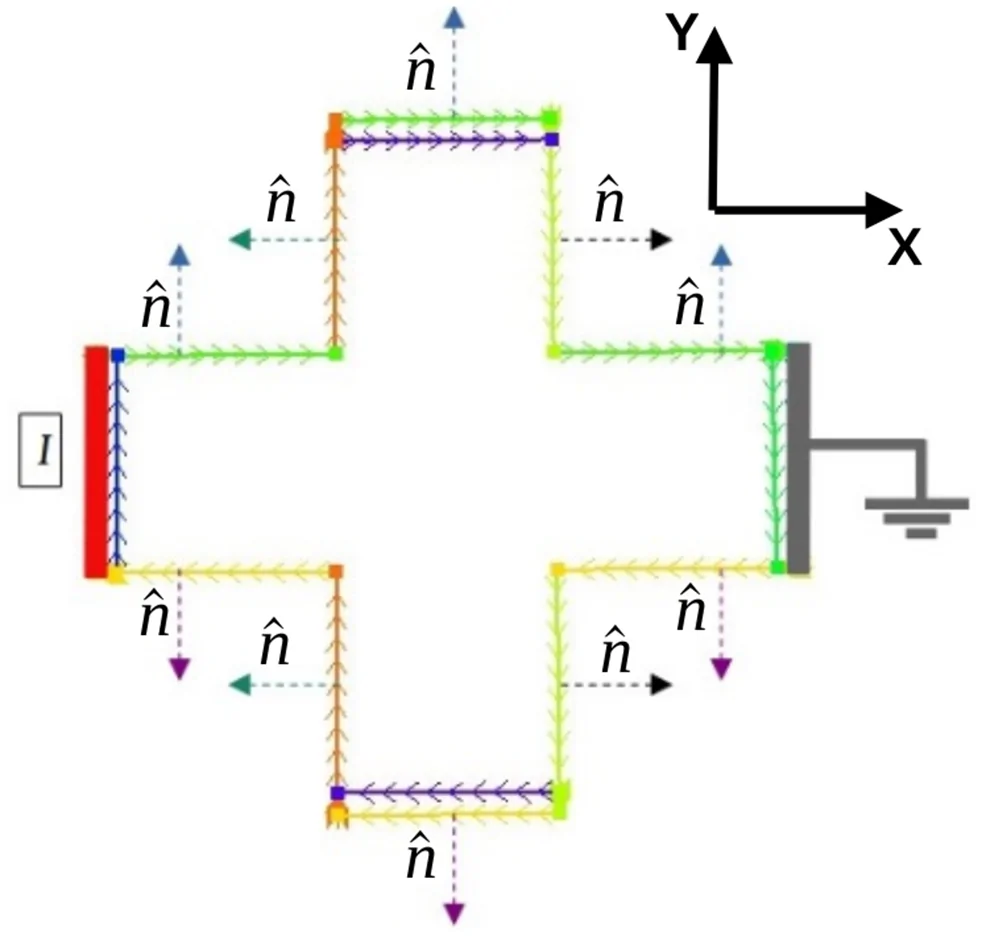
Boundary conditions on the cross-shaped graphene Hall sensor. Current is injected through the left vertical edge (red line), a 0 V potential is imposed on the right boundary (grey line) and *no-current flow* conditions are imposed on all the other boundaries.

For the Hall measurement configuration (see Fig 2), the right border (*x* = *L*/2) is set to 0 *V*, corresponding to a Dirichlet type condition. On the remaining boundaries, conditions on the current density are imposed and, therefore, related to the normal derivative of the potential (corresponding to Neumann boundary conditions).

At the current injection boundary (left border, (x=−L/2) see Fig 2), the Neumann condition is expressed as:


$$\begin{array}{c} \frac{\partial u}{\partial x}=\frac{1+(\mu B_{z}{)}^{2}}{ne\mu }\frac{I}{W}-\mu B_{z}\frac{\partial u}{\partial y} \end{array}$$ (9)

where, *n* is the effective carrier density given in Eq (7). Detailed explanations are given in the Appendix. On all the other boundaries (depicted in Fig 2), we assume that no current flows through the sample edges (*i.e.,* $\vec{J}\cdot \hat{n}=0$). This leads to the following boundary conditions:


$$\begin{array}{c} \frac{\partial u}{\partial x}=\pm \mu B_{z}\frac{\partial u}{\partial y} \end{array}$$ (10)

for the boundaries whose normal vector, $\hat{n}$, is parallel to the *x* axis ($J_{x}=0$), where the positive (negative) sign applies to the boundaries whose $\hat{n}$ points toward the negative (*resp.* positive) *x* direction.


$$\begin{array}{c} \frac{\partial u}{\partial y}=\pm \mu B_{z}\frac{\partial u}{\partial x} \end{array}$$ (11)

for the boundaries whose normal vector is parallel to the *y* axis ($J_{y}=0$),where the positive (negative) sign applies to the boundaries whose $\hat{n}$ points toward the positive (*resp.* negative) *y* direction. Notice that the physical model and boundary conditions are valid throughout all the regions, they are distinguished by the physical properties of each material, cf. Table-1). Solving the entire problem yields the spatial distribution of potential, *u*(*x*,*y*), over the Hall cross. The Hall voltage, $V_{H}$, is then extracted as the potential difference between the top and bottom boundaries and the Hall coefficient, $K_{H}$, is calculated using Eq. (2).

The vdP simulation is performed in the absence of magnetic field. In this measurement configuration, the boundary conditions are slightly modified compared to the Hall configuration (as illustrated in Fig 4): the current is injected in the left boundary, while the 0 *V* potential is imposed on the top boundary. The *vdP* voltage is then extracted between the right and bottom boundaries. This measurement is repeated for a second configuration in which the current injection and measurement contacts are rotated by 90°, as explained in the introduction. The vdP resistance, $R_{vdP}$, and the resistivity, $\rho_{2D}$, are then calculated using Eq. 5.

## 4 Results

Here, we examine the potential distribution *u*(*x*,*y*) over the Hall cross in both configurations to extract $V_{H}$ (and $K_{H}$) and $R_{vdP}$ (and ρ). First, the appropriate mesh size is determined and the model is validated for a perfectly homogeneous and isotropic device in which the analytical formula given by Eq. 2) is valid. We then extend the study by demonstrating the importance of including the four metallic contacts in the mesh when evaluating $V_{H}$. Next, we compare the Hall voltage obtained with FEM simulations with a more refined analytical formula [16]. Finally, we investigate the influence of geometrical defects (physical holes), without the geometrical restrictions demanded by the analytical models, to understand their impact on the sensor properties.

### 4.1 Mesh features

**Fig 3:**
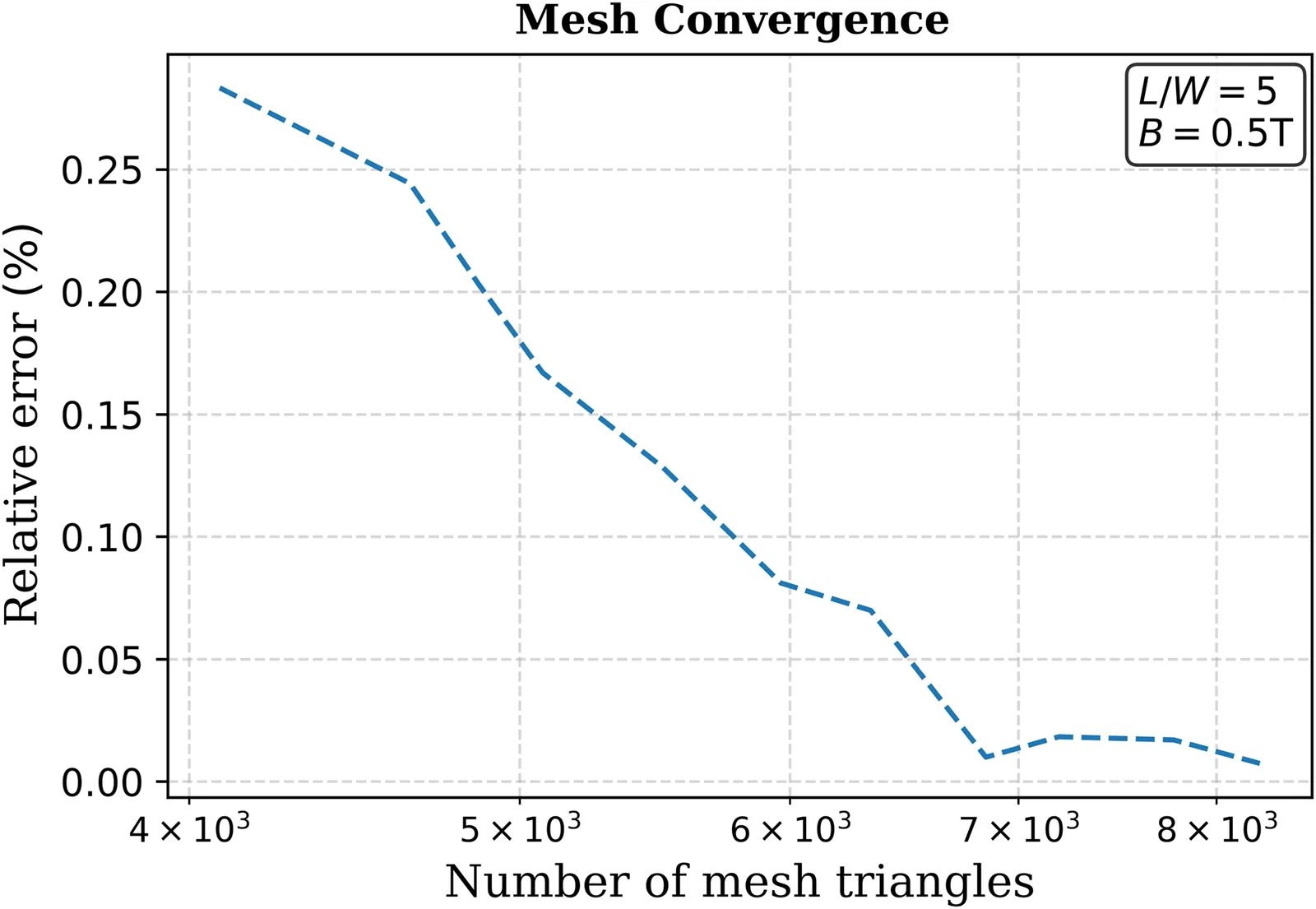
Mesh Convergence. Relative error of the simulated Hall voltage as a function of the number of mesh triangles for a length-over-width ratio *L*/*W* = 5 under an applied magnetic field *B* = 0.5 T.

The mesh quality is crucial for the accuracy of the numerical simulation. To determine an appropriate mesh size, a convergence study is performed to determine the acceptable range (the mesh of the Hall-cross is illustrated on (Fig 1.a)). From the convergence plot of Fig 3 it can be inferred that, for a mesh number of about 7 000, the relative error (≈0.01%) can be considered to be negligible.

In the optimal mesh configuration (namely 7 000 finite elements) the computational time is about 0.4 *s*. It should be noticed that the presented numerical simulations were performed on a laptop equipped with an Intel Core i7 CPU running at 2.70 GHz operating on a 64-bit Windows system. Therefore, the simulation does not require high-performance computing clusters or extensive memory resources, making it easily accessible for both research and educational purposes.

### 4.2 Validity of the FEM model

The preliminary step in validating the formalism and geometry is to compare it with a reference model. For this purpose, the resistivity and the Hall voltage as a function of magnetic field is obtained with the analytical and Fem models for comparison. The numerical values are obtained for the vdP and Hall configurations of a graphene Hall cross with gold contacts at $V_{g}=0$ V, *i.e.,* the typical residual doping of $n^{*}$. The analytical value of the graphene resistivity ρ is given by Eq. 3, and the Hall coefficient $K_{H}$ is given by Eq. 2. summarized in Table 1. As mentioned in the introduction, in the case of graphene, electron and holes will coexist near the Dirac point and the one-carrier model presented here will be limited to high gate voltages. For the parameters in Table 1, this would correspond to $V_{g}<-1.4$ V for holes and $V_{g}>4.2$ V in the case of high quality devices at room temperature.

**Table 1:** Simulation parameters.

Parameters	Value	Unit
Graphene mobility (μ)	10^4^	*cm*^2^/*V s*
Graphene residual doping ($n^{*}$)	10^11^	*cm* ^-2^
Gate capacitance ($C_{g}$)	$1.15\times 10^{-8}$	*F*/*cm*^2^
Gold mobility ($\mu_{m}$)	40	*cm*^2^/*V s*
Gold carrier doping ($n_{m}^{*}$)	10^18^	*cm* ^-2^
Length, (*L*)	3	*mm*
Width of the metallic contacts ($L_{m}$)	0.1	*mm*
Bias current (*I*)	1	*mA*

Table notes: The gate capacitance is typical for a backgate with a ~300 nm thick *SiO*_2_ dielectric. Gold carrier density is $n_{m,3D}^{*}=10^{29}m^{-3}$, and is calculated for this model for a 100 *nm* thick contact. The length *L* is maintained constant throughout this paper, *W* may vary.

**Fig 4:**
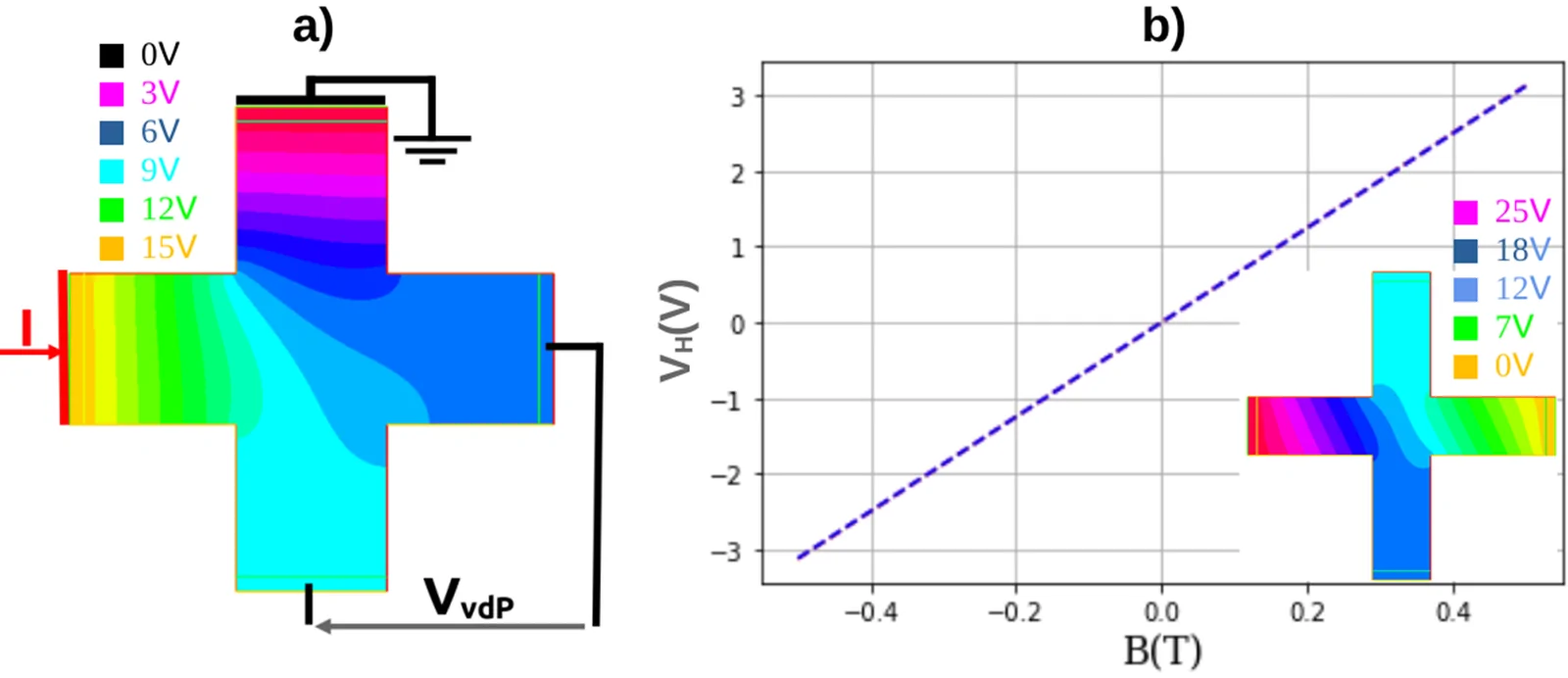
Validity of the model in the perfect case. (a) Illustration of the potential distribution in a graphene Hall cross in the vdP measurement configuration (*L*/*W* = 3). (b) Simulated and calculated Hall voltage using Eq (2) as a function of magnetic field for a thin Hall cross (*L*/*W* = 5, other parameters are given in Table 1). The inset Fig shows the potential distribution in the thin Hall cross at *B* = 0.5 *T*.

The potential distribution, *u*(*x*,*y*), in the vdP configuration is shown in Fig 4.a. *u*(*x*,*y*) and the corresponding voltage, $V_{vdP}$, obtained in this configuration, as welle as in the rotated configuration, are identical since the device is isotropic and symmetric. The vdP resistance, $R_{vdP}$, and the resistivity, $\rho_{vdP}$, are then obtained by applying Eq (5). The comparison between the analytical value (ρ in Eq (3) and the one from the simulation shows a very good agreement, with a relative error below 0.1%.

Next, the Hall effect is simulated in the same geometry and is compared with the Hall voltage obtained with the analytical model given by Eq (2). For this comparison, we consider a thin Hall cross close to the ideal case with an aspect ratio *L*/*W* = 5 which allows the shape effect to be neglected, as discussed in section 4.4 and in Ref. [14]. This comparison between the simulated and calculated Hall voltages is depicted in Fig 4.b, with *u*(*x*,*y*) displayed in the inset. There is almost no variation of the potential distribution in graphene close to the contacts, and $V_{H}$ depends linearly on magnetic field, as expected. The two values show a very good agreement, with a relative error below 0.1%.

Following the validation of the FEM simulation, it can be used to investigate various effects in more realistic devices: the influence of metallic contacts, and the geometrical aspect ratio.

### 4.3 Effect of metallic contacts on the potential distribution

**Fig 5:**
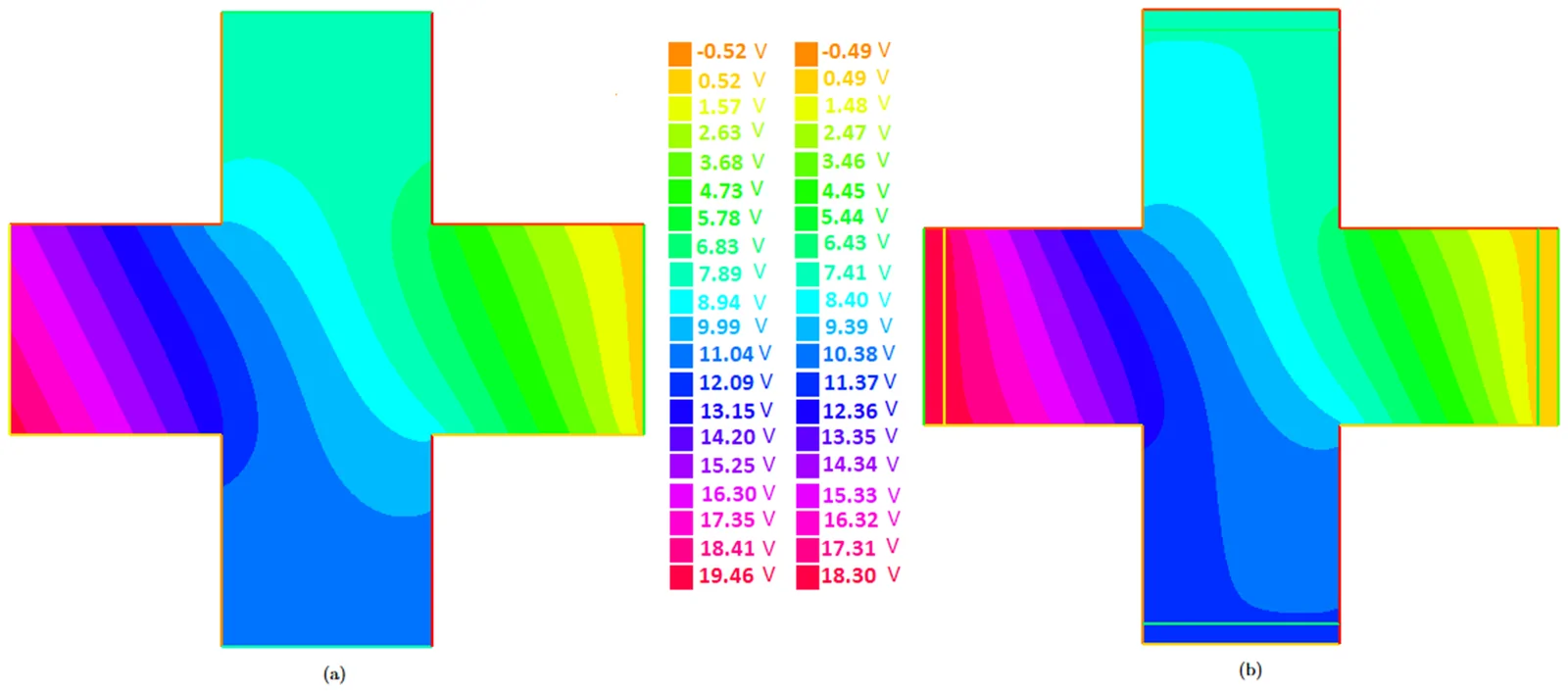
Role of the metallic contacts on the potential distribution. Hall cross with graphene only (left), and with graphene and four metal contacts (right). The simulation is performed under a magnetic field, *B* = 0.5 *T* and a fixed gate voltage, $V_{g}=0\;V$. The remaining simulation parameters are detailed in Table 1.

We take advantage of the versatility of the simulation to illustrate the effect of the metallic contacts on the potential distribution by performing simulations with and without contacts, as shown in Fig 5. At this point, it is worth noticing that, we implemented Neumann boundary condition which is closer to the experimental conditions even if Dirichlet conditions would be enough to homogenize the potential at the metalized edges (current injection and floating contacts). The main drawback of using Dirichlet condition would be to compute the current after calculation.

On the left of Fig 5 (without metallic contacts), the equipotential lines are inclined near the current injection and grounded edges, and they are not uniform (horizontal) across the two measurement arms (top and bottom). On the right Fig 5 (with metallic contacts), it is obvious that the metallic contacts homogenize (weak color gradient) the potential near the current injection region (left branch), and near the Hall voltage contacts (top and bottom edges). This artifact in the model without metallic contacts is due to the boundary conditions and the relatively low conductivity of graphene compared with gold (around 4 orders of magnitude difference for a reasonable gold thickness). Metallic contacts tend to homogenize the potential over a very short distance, even in the regions where the current enters or leaves the contacts.

A quantitative comparison of the Hall voltage value obtained from the geometry with and without contacts gives a relative error of ~3.3% for the Hall voltage calculated without contacts. This highlights that the presence of metallic contacts significantly modifies the potential distribution within the cross, and a large underestimation of the Hall voltage could be introduced by neglecting them. Taking care of such artifacts is thus essential for reliable simulations.

### 4.4 Shape effect

In real devices, two effects will modify the sensor response. First, the aspect ratio will modify the potential distribution and, therefore the Hall voltage. This geometrical effect is accounted for by a correction term, called the shape factor, *G*, introduced into the analytical model [16]. Second, the carrier scattering processes in the measured material may also reduce the Hall coefficient and are acccounted for through the Hall scattering factor, $r_{H}$. The analytical expression for the Hall voltage, Eq. 2, for a 2D material, becomes:


$$\begin{array}{c} V_{H}=r_{H}\;G\frac{IB}{nq} \end{array}$$ (12)

In this study, we assume that $r_{H}\approx 1$, as is typically observed in graphene based devices. More details about the Hall scattering factor in graphene are discussed in section 4.6 and in the appendix (3.6).

**Fig 6:**
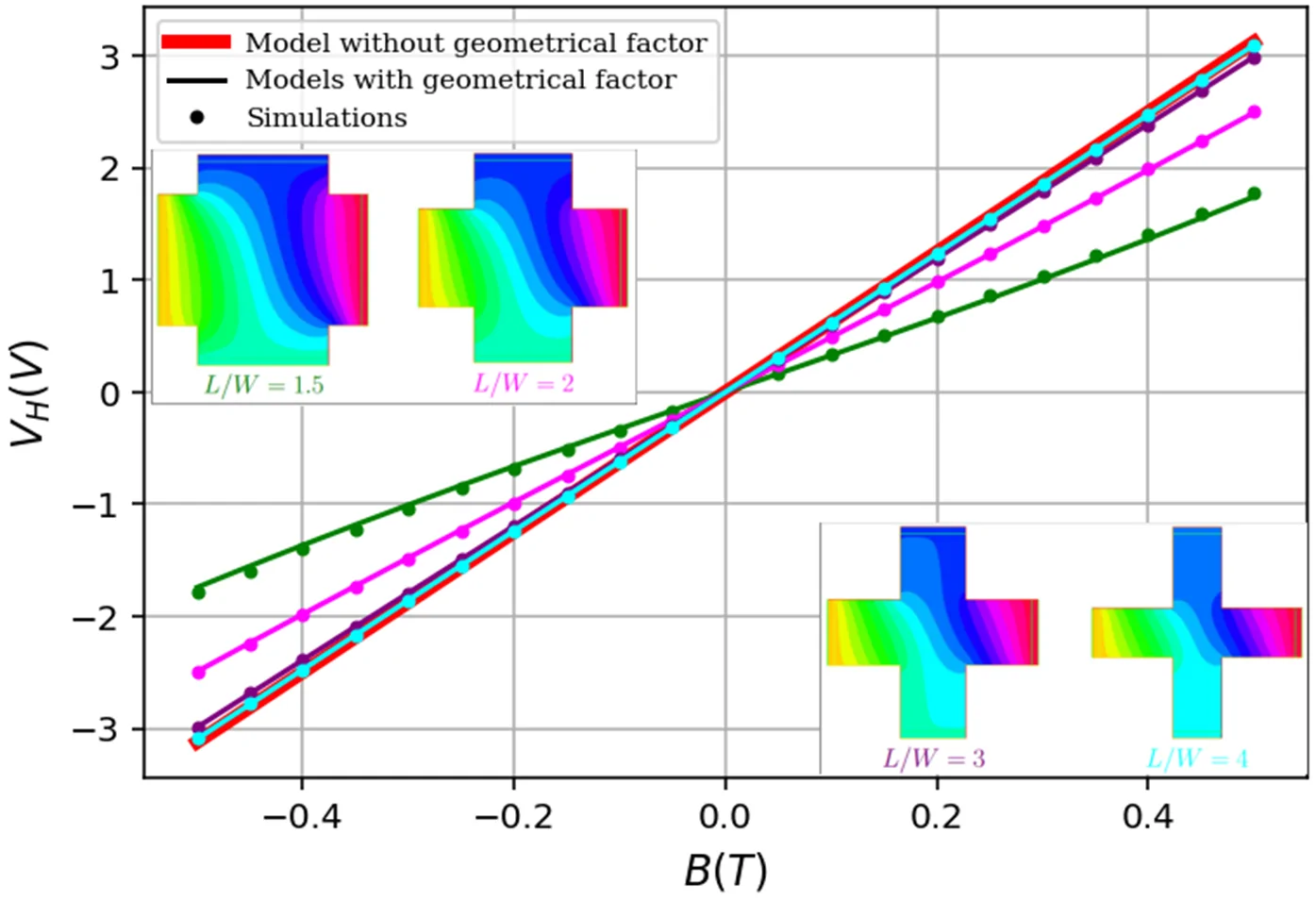
Hall voltage $V_{H}$ as a function of magnetic field *B* for different *L*/*W* ratios. The gate voltage is fixed gate Voltage $V_{g}=0$. The other simulation parameters are listed in Table 1. The inset figures show the potential distribution in the thin Hall cross at *B* = 0.5 *T.*

For a Hall cross, the geometrical correction factor is given by [16]:


$$\begin{array}{c} G=1-5.0267\frac{\theta_{H}}{\tan(\theta_{H})}\exp(-\frac{\pi }{2}\frac{L}{W}) \end{array}$$ (13)

where $\theta_{H}=\mu_{g}B\;$ is the Hall angle [20]. The FEM simulations were performed for various geometrical configurations (*L*/*W* = 4, *L*/*W* = 3, *L*/*W* = 2, *L*/*W* = 1.5, cf. inset of Fig 6). The simulated Hall voltages were then compared with the analytical predictions obtained using Eq. 13 for each aspect ratio (Fig 6). It can be noticed that $V_{H}$ decreases when the width approaches the length (when *L*/*W* decreases). The relative error between the simulated and analytical model (summarized in Table 2) demonstrates that the analytical model accurately captures the effect of the geometry for the larger *L*/*W* ratio (where the geometrical corrections are small) but becomes less accurate as *L*/*W* decreases.

**Table 2:** Maximum relative error $\epsilon_{r}=\frac{V_{H,an}-V_{H,sim}}{V_{H,an}}$ between the analytical model including the geometrical correction factor Eq (13) (with $r_{H}=1$) and the simulated voltage for different *L*/*W* ratios.

*L*/*W*	1.5	2	3	4
** $\max(\epsilon_{r})(\%)$ **	3.972	0.814	0.146	0.162

For L/W≥3, the maximum relative error is sufficiently small to be considered negligible. This justifies the choice of *L*/*W* = 5, where the geometrical factor is close to unity, for validating the FEM model with the simplest analytical expression. Conversely, *L*/*W* = 3 provides a more suitable geometry to evidence the role of metallic contacts. In the following section, we therefore use *L*/*W* = 3 to clearly illustrates the influence of a geometrical defect as a function of its position.

### 4.5 Defect effect

The advantage of the simulation is illustrated through a case that is both potentially detrimental to a sensor performance and not accessible through a straightforward analytical derivation. The dependence of the Hall voltage, $V_{H}$, its offset, $V_{Offset}$, the vdP voltage, $V_{vdP}$, and the resistivity, ρ, as a function of a defect position are studied. Indeed, geometrical defects can influence both $V_{H}$ and $V_{vdP}$. Consequently, the accuracy of the resistivity, carrier density and mobility extracted using these methods for material characterization can be strongly affected. Moreover, the performances for magnetic field sensing will also be degraded.

In the case of magnetic sensing, a defect induces an offset voltage which limits the dynamic range and increases the low frequency (LF) noise (by shifting the cut-off frequency to a regime where the low frequency noise dominates). Ultimately, the LF noise degrades the sensor resolution. This signature is even used to study the fabrication process quality of commercial Hall sensors [21]. It has been shown for graphene [22] that the LF noise depends on various parameters, including mobility inhomogeneity and carrier density fluctuations. A complete quantification requires characterization of the graphene material and its interfaces. Here, we demonstrate that the FEM simulation tool has the potential to study the effect of a small defect and the importance of its position in the device.

**Fig 7:**
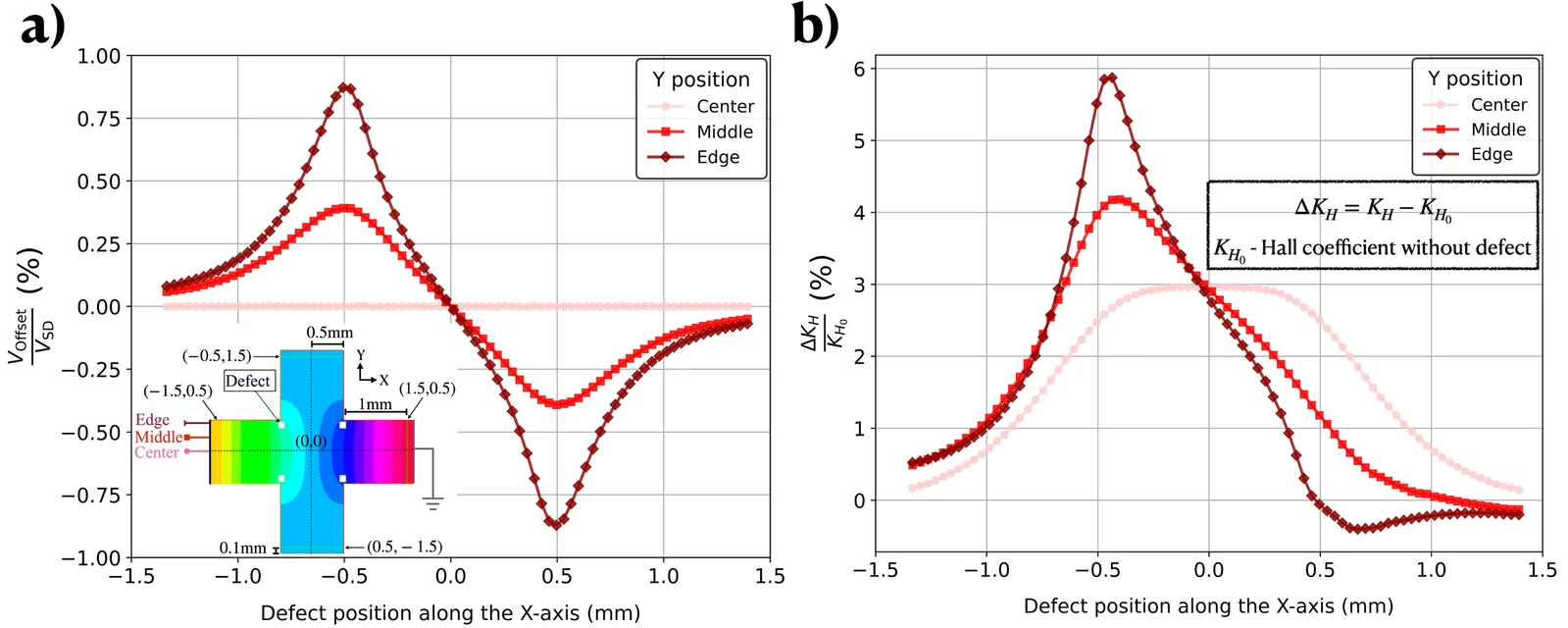
Simulated offset voltage and Hall coefficient for a Hall cross geometry with an aspect ratio *L*/*W* = 3 containing a single square defect of size *d* = *W*/15. (a) Relative variation of the offset voltage with respect to the source–drain voltage, ($\frac{V_{offset}}{V_{SD}}$), as a function of the defect position along the *X*-axis for three fixed defect positions along the *Y*-axis: center (Y=0mm), middle (Y=0.25mm), and edge (Y=0.375mm), as indicated in the inset. The inset also highlights the four defect locations corresponding to the maximum offset voltage. Simulations were performed using the parameters listed in Table 1, with $V_{g}=0\;V$ and B=0T.(b) Relative variation of the Hall coefficient, $\frac{\Delta K_{H}}{K_{H_{0}}}$, as a function of the defect position along the *X*-axis for the same *Y*-positions as in (a), simulated at $V_{g}=0\;V$ and B=0.5T.

First, a small square defect of size (*d* = *W*/15) (white square in Fig 7.a) is introduced into a Hall cross with *L*/*W* = 3 and *L* = 3 mm. The Hall effect is then simulated by moving the defect along the X-axis (current injection direction) while considering three positions along the Y-axis: near the device edge (*Y* = 3*W*/8), at the center (*Y* = 0) and between these 2 positions (*Y* = *W*/4). As expected, the equipotential lines of *u*(*x*,*y*) are distorted around the defect as shown in Fig 7.a. This distortion gives rise to an offset voltage, $V_{Offset}=V_{H}(B=0)$, which is added to the Hall voltage defined in Eq (12).

The dependence of $V_{Offset}$ on the defect position is plotted in Fig 7.b. It can be observed that a defect located at the center of the cross (*X* = 0) or moving along the center of the arm (*Y* = 0, light pink line) does not induce any offset. As soon as the defect is displaced from the center position, X≠0, a finite offset voltage appears, $V_{Offset}\neq 0$. This offset is antisymmetric with respect to the center of the cross, with $V_{Offset}>0$ on the current injection side (*X* < 0) and $V_{Offset}<0$ on the grounded side (*X* > 0). As the defect gets closer to the edge (increasing *Y*), the offset increases. In all cases, the maximum offset is reached when the defect is aligned with the edges of the voltage measurement arms (X=±W/2) As a consequence, a defect placed near a corner produces the largest perturbation of the potential lines in the measurement arms, and therefore the largest offset voltage contribution (inset of Fig 7.b).

The simulations also reveal that the presence of a defect modifies the Hall coefficient, $K_{H}$, compared to its expected analytical value (Fig 7.c). A relative error $\frac{\Delta K_{H}}{K_{H0}}=\frac{K_{H}-K_{H0}}{K_{H0}}$ as high 6% can be reached for the position closest to the corner of the Hall cross (X=±W/2 and *Y* = 3*W*/8), corresponding to the one giving the highest offset. More generally, a defect induces a relative error between 2 and 6% when it is located within the central region of the Hall cross. Even for *Y* = 0, where no Hall voltage offset is observed, the Hall coefficient still exhibits a relative error of approximately 3%. Unlike the offset voltage, the variation in ($K_{H}$) is therefore not symmetric with respect to the center position. This behavior can be attributed to a stronger distortion of the potential lines when the injected current is deviated toward the voltage measurement arms. As a result, this type of simulation shows that even a small defect can have a much stronger effect on the measured Hall coefficient compared to geometrical effects. This can be significantly degrade a device sensitivity or an accurate charge density determination in material characterization.

**Fig 8:**
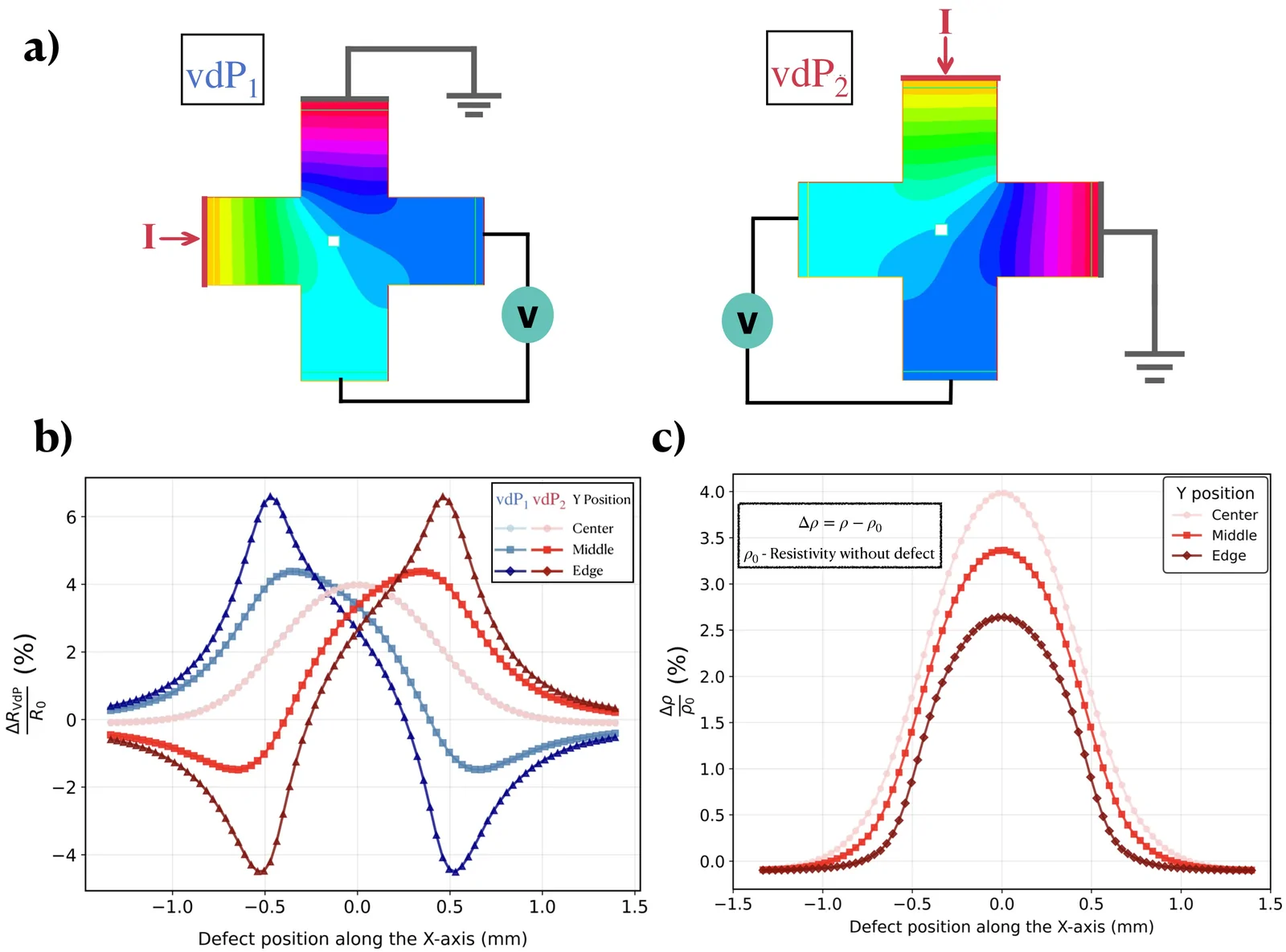
Simulated van der Pauw (vdP) potential of the Hall-cross geometry with a single defect. (a) Potential distribution for the two vdP measurement configurations used in (b) (${vdP}_{1}$ and ${vdP}_{2}$) with the defect located at the centre of the Hall cross (coordinates (0,0)). (b) Relative variation of the simulated vdP resistance with respect to the defect-free resistance $\frac{R_{vdP}-R_{0}}{R_{0}}=\frac{\Delta R_{VdP}}{R_{0}}$, as a function of the defect position along the X-axis for the fixed Y-positions, center (*Y* = 0 mm), middle (*Y* = 0.25 mm), and edge (*Y* = 0.375 mm). (c) Relative variation of the simulated resistivity with respect to the defect-free value, expressed as $\frac{\Delta \rho }{\rho_{0}}$, for the same defect positions as in (b). All the simulations were performed at $V_{g}=0V$ and B=0T.

Let us now consider resistivity characterization using the van der Pauw method. From the vdP voltage measurement (the electrical configuration is illustrated in Fig 8.b), we derive the two resistances $R_{vdP1}$ and $R_{vdP2}$. The resistivity is deduced by averaging the two vdP resistances ($R_{vdP}=(R_{vdP1}+R_{vdP2})/2$) and applying Eq (5). An isolated defect also has an impact on the measurement and consequently affects the validity of the vdP method for characterizing the material resistivity. For this study, the simulation is performed with a defect moving along the X-axis at the same three *Y* positions, as in the previous case, and for the two rotated configurations $V_{vdP1}$ and $V_{vdP2}$. Usually $V_{vdP1}$ and $V_{vdP2}$ compensate each other in a fairly homogeneous device (Eq (4) and Eq (5)). Contrary to the Hall offset case, the deformation of the potential lines in Fig 8.a is not sufficient to anticipate the effect, even in the least favorable position ((*X*,*Y*) = (0,0)).

Looking at the relative error on the measured vdP resistance (cf. Fig 8.b), it is observed that all the positions induce an error either on $V_{vdP1}$ or $V_{vdP2}$. The problem is fully symmetric with respect to *X* = 0 between the two configurations, since the defect moves from the current injection region toward the first measurement arm in *vdP*1, and from the second measurement arm toward the grounded arm (current collection) in *vdP*2.

For a position near the edge, and *vdP*1, the error increases from 1% at the current injection edge (X=−1.5 mm) and reaches a maximum of ~6% at the edge of the grounded arm (X=−W/2=−0.5 mm), then decreases to ~2% at the center. Moving toward one of the measurement arms, the error becomes 0% and reaches another (absolute) maximum of ~4% at the next edge (*X* = *W*/2 = 0.5 mm). For a defect centered at *Y* = 0, the error is always positive, starting from 0% (X=−1.5 mm), and reaches a maximum of 4% at the center; then it symmetrically decreases. The intermediate case exhibits a mix of both configurations.

Hence, for a given position of the defect, averaging the two measurements will give an error in all the cases (cf. Fig 8.c). The error $\frac{\Delta \rho }{\rho_{0}}$ starts from 0% at the contacts and increases until reaching a maximum in the central part, |*X*| < *W*/2 (as illustrated in Fig 8). When the defect is near the edge, partial compensation still occurs with $\frac{\Delta \rho }{\rho_{0}}⩽2.5\%$ and an error of 2% at X=±W/2, although this corresponds to the least favorable case for the Hall effect (a defect in a corner). When the defect moves along the center, although the effect is weak near the contacts, it does not fully compensate and a maximum error of $\frac{\Delta \rho }{\rho_{0}}=4\%$ is reached at *X* = 0, although this case corresponds to $V_{Offset}=0$ and $\frac{\Delta K_{H}}{K_{H0}}=3\;\%$. This worst error after correction is obtained for a defect at the center of the cross ((*X*,*Y*) = (0,0)), since it creates the strongest asymmetry in the potential lines for this non-local measurement with a 90° angle between source and drain (and between the measurement arms), similarly to $K_{H}$.

### 4.6 Comments and discussion

These results show that proper finite element modeling of a Hall cross not only reproduces effects that can be predicted analytically, but also predicts more complex and less intuitive cases. This is illustrated here by a small geometrical defect in the structure. This FreeFEM++ script simulation can be adapted to any sensor geometry and to any size and shape of defect. It can even be extended to 3D material. It provides a powerful tool for interpreting measurements or validating analytical models.

More realistic devices can now be easily modeled, and the expected results can be predicted by introducing the exact device geometry and even physical holes in the structure, which are easily identified and measured by optical imaging. This is of particular interest when the material properties are not perfectly known, and when resistivity and Hall measurements are used for material characterization. This FEM model should prove to be useful for accurate magnetotransport studies by correcting measurements to extract accurate values of charge density and mobility, and to avoid limitations in precision arising from analytical approximations. Further work can be done to include material anisotropy, or, in the specific case of graphene, we plan to take into account the co-existence of electrons and holes owing to thermal excitation and device inhomogeneities [17]. This is of particular interest since, for this zero bandgap semiconductor, the maximum sensitivity (and Hall coefficient) is governed by this phenomenon. Moreover, a correction of $r_{H}≃4$ to the Hall coefficient $K_{H}$ (Eq. 12) is expected theoretically at very small doping because of scattering, although this has not been observed experimentally because of the co-existence of electron and holes at such carrier density (Appendix 3.6). It would then be of prime interest to account for different microscopic scattering mechanisms and introduce them in the two-carrier model to fully reproduce experimental results.

Finally, this model is also useful for device engineering. In particular, even if the offset in $K_{H}$ can be corrected by averaging the two possible measurement configurations, some discrepancies in the Hall voltage can remain (as illustrated by averaging positive and negative values of the position X in Fig 7). More importantly, the main contribution to the 1/*f* noise comes from this offset voltage and would limit the minimum detectable field. More generally, these simulations will be a powerful tool to optimize contact shape and size to maximize sensitivity [16].

## 5 Conclusion

We have described a FEM model of the Hall effect and vand der Pauw measurements in cross-shaped graphene devices using FreFEM++. The formulation of the weak form leads to a physical equation indicating the absence of explicit spatial dependence on the magnetic field. This dependence appears in the boundary conditions, since charges accumulate at the edges of the Hall cross. The FEM model has been validated through comparisons with analytical equations, including shape (form factor) effects. We have shown that current injection and voltage measurements closer to experimental conditions are obtained by including the metallic contacts in the model. Finally, we have shown that simulations reproduce realistic conditions which cannot be reproduced by analytical approximations and enable the estimation of the associated errors. The presence of a defect can modify both the vdP and the Hall voltages, and the resulting measurement error can be corrected using our model.

This work can be used for educational purposes, but can also be adapted for device optimization and material characterization. This Hall effect simulation can be readily adapted to any sensor geometry, defect size and position, and can be extended to investigate other cases such as anisotropic materials, two-carrier models, or geometry optimization. Similarly, accurate electronic properties of materials can be extracted by appropriate corrections based on such FEM model.

## Supporting information

S1 File Hall effect FreeFEM++ simulation script. This file is a commented FreeFEM++ script which can be ran on a PC after installing FreeFEM++. The simulation results presented in the paper can be reproduced using the script. The script can be modified by anyone to adapt it. For convenience, the sript is saved in .txt file, to run it you will have to replace the txt extension by .edp.
(ZIP)

S1 Appendix Physical description of the Hall effect. In this appendix, details are given on the calculations and assumptions to derive the differential equation, its weak form and the boundary conditions.
(PDF)

S2 Appendix Scattering factor in graphene. (PDF)
