# Supplementary material for: Hall effect FEM modeling using FreeFEM++: Illustration on a cross-shaped graphene sensor
Source: PLoS One. 2026 Sep 24;21(9):e0357905. doi: 10.1371/journal.pone.0357905 (PMC13607873; doi:10.1371/journal.pone.0357905)
Supplement: Supplementary file 1 [file pone.0357905.s002.pdf]

## S1 Appendix Physical description of the Hall effect

In this appendix, details are given on the calculations and assumptions to derive the differential equation, its weak form and the boundary conditions.

**PDE:**

The fundamental equations of electrodynamics under the stationary regime, i.e. $\frac{dn}{dt} = 0$ are given by Eqs.(1) to (4)):

$$\text{Electric field:} \quad \vec{E} = -\vec{\nabla} u \tag{1}$$

$$\text{Continuity equation:} \quad \vec{\nabla} \cdot \vec{J} = 0 \tag{2}$$

$$\text{Current density:} \quad \vec{J} = nq\vec{v} \tag{3}$$

$$\text{Lorentz Force:} \quad \vec{F} = q(\vec{E} + \vec{v} \times \vec{B}) \tag{4}$$

where,$\vec{v}$ is the velocity of the charge carrier $q$ (electron or hole) and $\vec{E}$ is the electric field due to the potential $u(x, y)$. Assuming that $\vec{F} = md\vec{v}/dt = \vec{v}/\tau$ and substituting $\vec{v}$ by $\vec{J}$ thanks to Eq (3), Eq (4) becomes :

$$\vec{J} = \frac{nq^2\tau}{m} \left( \vec{E} + \frac{\vec{J}}{nq} \times \vec{B} \right) \tag{5}$$

where, $\tau$ is the recombination or the mean free time and $m$ is the mass of the charge carrier. In the 2D regime [1], the current density components are obtained by expanding Eq (5) and using Eq (1) with the magnetic field applied along the z-direction.

$$J_x = \frac{-nq\mu}{1 + (\mu B_z)^2} [\frac{\partial u}{\partial x} + \mu B_z \frac{\partial u}{\partial y}] \tag{6}$$

$$J_y = \frac{-nq\mu}{1 + (\mu B_z)^2} [\frac{\partial u}{\partial y} - \mu B_z \frac{\partial u}{\partial x}] \tag{7}$$

where, $\mu = \frac{q\tau}{m}$ is the charge carrier mobility. In the one-carrier regime, the gate and spatial dependent charge carrier density, $n(x, y)$, is modeled through capacitive coupling [ [**?**]],

$$n(x, y) = \frac{C_g}{q} (V_g - u(x, y)) + n^* \tag{8}$$

where, $C_g$ is the capacitance between the one-carrier system (graphene or any semiconductor) and the Si/metallic electrode through the dielectric layer (typically $SiO_2$), and $n^*$ is the charge carrier density at $V_g = 0V$ (doping).

Substituting the current density vector components (Eq (6) & Eq (7)) in the continuity equation Eq (2), under the assumption that the electrical conduction is only due to holes, gives the following matrix equation:

$$\vec{\nabla} \cdot \vec{J} = \begin{pmatrix} \frac{\partial}{\partial x} & \frac{\partial}{\partial y} \end{pmatrix} \begin{pmatrix} \frac{ne\mu}{1+(\mu B_z)^2} & \frac{ne\mu^2 B_z}{1+(\mu B_z)^2} \\ \frac{-ne\mu^2 B_z}{1+(\mu B_z)^2} & \frac{ne\mu}{1+(\mu B_z)^2} \end{pmatrix} \begin{pmatrix} \frac{du}{dx} \\ \frac{du}{dy} \end{pmatrix} = 0 \tag{9}$$

[1] The thickness of the conducting channel and the distance between the gate dielectric and the conducting channel are negligible compared to other lengths

which can be identified to Ohm's Law:

$$\vec{J} = \sigma \vec{E} \implies \vec{J} = -\sigma \vec{\nabla} u \tag{10}$$

suggesting that $\sigma$ is a rank 2 tensor with magnetic field and carrier density dependencies.

Next, the continuity equation is expressed by Eq (2). By substituting the current density given by Ohm's law (Eq (10), and using the conductivity tensor(Eq (9), we obtain:

$$\vec{\nabla}.(\sigma \vec{\nabla} u) = 0 \tag{11}$$

By substituting $n$ using Eq (8), we derive the Partial Differential Equation (PDE) to be solved:

$$\boxed{\frac{\mu C_g}{(1+(\mu B_z)^2)}[((u - V_g) + \frac{en^*}{C_g})\nabla^2 u + (\frac{\partial u}{\partial x})^2 + (\frac{\partial u}{\partial y})^2] = 0} \tag{12}$$

It is noteworthy that the magnetic field dependence appears in the coefficient of the above differential equation, which is equated to zero. Therefore, the magnetic field dependence does not appear explicitly in the PDE of Eq (12).

**Variational form:**

To solve a differential equation over a given geometry in FreeFEM++, it must first be expressed in its variational form (weak or integral form). This is obtained by integrating the equation over the surface and expanding it using integration by parts. Thus, after some calculation, the variational form of Eq 12 is derived as follow:

$$\begin{aligned}\Bigg[\int_{\partial S} \frac{\mu C_g}{1+(\mu B_z)^2}\left(\frac{en^*}{C_g} - V_g\right)\vec{\nabla} u \cdot \hat{n}\, v\, dl \\ - \iint_S \frac{\mu C_g}{1+(\mu B_z)^2}\left(\frac{en^*}{C_g} - V_g\right)\vec{\nabla} u \vec{\nabla} v\, dS \\ + \iint_S \frac{\mu C_g}{1+(\mu B_z)^2}\left(u\,\nabla^2 u + \left(\frac{\partial u}{\partial y}\right)^2 + \left(\frac{\partial u}{\partial x}\right)^2\right) v\, dS\Bigg] = 0\end{aligned} \tag{13}$$

where, $v$ is a test solution, $dl$ and $dS$ are the geometry's infinitesimal displacement and surface respectively along the boundaries $\partial S$ and over the surface $S$. To get rid of the $2^{nd}$ degree terms ($\frac{\partial u}{\partial x}^2$ and $\frac{\partial u}{\partial y}^2$), the term with "$u\,\nabla^2 u\, v$" can be further simplified as shown below:

$$u(\nabla^2 u)v = \vec{\nabla} \cdot (uv\vec{\nabla} u) - \vec{\nabla} u \cdot \vec{\nabla} u\, v - u\vec{\nabla} u \cdot \vec{\nabla} v \tag{14}$$

Further simplification, using the divergence theorem, gives the intended variational form with $1^{st}$ degree differential terms, which depends only on the potential and the magnetic field.

$$\boxed{\begin{aligned}\Bigg[\int_{\partial S} \frac{\mu C_g}{1+(\mu B_z)^2}\left(\frac{en_0}{C_g} - V_g\right)(\vec{\nabla} uv + uv\,\vec{\nabla} u) \cdot \hat{n}\, dl \\ + \iint_S \frac{-\mu C_g}{1+(\mu B_z)^2}\left(\frac{en_0}{C_g} - V_g\right)\vec{\nabla} u \cdot \vec{\nabla} v\, dS \\ + \iint_S \frac{-\mu C_g}{1+(\mu B_z)^2} u\,\vec{\nabla} u \cdot \vec{\nabla} v\, dS\Bigg] = 0\end{aligned}} \tag{15}$$

The second and third terms correspond to the variational form of the Laplace operator, and the non-linear term originates from the local potential variation of the charge carrier density given by Eq (8). The first integral term represents the boundary condition of the problem, which naturally originates from the derivation of the variational form.

In solving the PDE with FreeFEM++, the non-linear terms are neglected. Thus, the weak form of the PDE simply becomes:

$$\iint_S \frac{-\mu C_g}{1+(\mu B_z)^2}\left(\frac{en^*}{C_g}-V_g\right)\vec{\nabla}u\cdot\vec{\nabla}v dS-\int_{\partial S}\frac{-\mu C_g}{1+(\mu B_z)^2}\left(\frac{en^*}{C_g}-V_g\right)\vec{\nabla}u\cdot\hat{n}\,v\,dl=0 \tag{16}$$

**Boundary conditions:**

The writing of the appropriate boundary conditions is essential to describe the physical problem to be solved. As discussed above, the magnetic field dependence does not arise from the PDE of the physical problem but indeed comes from the boundary conditions. We recall the two types of boundary conditions used in this problem :

- **Dirichlet Condition:** $u(a) = g$
- **Neumann Condition:** $\vec{\nabla}u \cdot \hat{n} = f$

where $g$ and $f$ are scalar functions defined over a boundary $S$. To determine the appropriate form of the Neumann boundary condition in the Hall cross, physically plausible conditions are imposed to the current density component normal to each boundary. On the current-injection boundary, it is fixed to the injected current density, whereas on all other boundaries it is set to zero.

Making use of this in Eq (6) & Eq (7) we derive the Neumann boundary conditions. On the boundaries whose normal vector is towards the $+x$ axis (see Fig **??**), the condition is deduced from $J_x = 0$ as follows (for boundaries oriented towards the $-x$ axis, it will be opposite):

$$\frac{\partial u}{\partial x} = -\mu B_z \frac{\partial u}{\partial y} \tag{17}$$

Similarly for boundaries whose normal vector is towards the $y$ axis (see Fig **??**), the condition is deduced from $J_y = 0$ as follows (for boundaries oriented towards the $-y$ axis it will be opposite):

$$\frac{\partial u}{\partial y} = \mu B_z \frac{\partial u}{\partial x} \tag{18}$$

Finally, we express the boundary condition on the boundary where the current ($I$) is injected. In the configuration studied here, this the left boundary (see Fig **??**) and a positive current corresponds to a current density vector directed along the $-x$ direction. Thus $\vec{J}\cdot\hat{n} = -J_x n_x$. Assuming an homogeneous current density along this boundary, $J_x = I/W$ and is oriented towards the $-x$ axis. The boundary condition is expressed as:

$$\frac{\partial u}{\partial x} = \frac{1+(\mu B_z)^2}{ne\mu}\frac{I}{W} - \mu B_z \frac{\partial u}{\partial y} \tag{19}$$

Finally, we apply a Dirichlet $u = 0$ condition on the grounded boundary.
