# Supplementary material for: Hall effect FEM modeling using FreeFEM++: Illustration on a cross-shaped graphene sensor
Source: PLoS One. 2026 Sep 24;21(9):e0357905. doi: 10.1371/journal.pone.0357905 (PMC13607873; doi:10.1371/journal.pone.0357905)
Supplement: Supplementary file 2 [file pone.0357905.s003.pdf]

S2 Appendix. Scattering factor in graphene :

The Hall factor, r_H, is a dimensionless parameter representing the ratio between the Hall mobility, μ_H (determined experimentally), and the conductivity mobility of the material μ. It depends on both the carrier scattering mechanisms and the band structure of the material [[**?**]] .

For semiconductors (n-type or p-type) with a parabolic dispersion band:

$$r_H = \frac{\langle \tau^2 \rangle}{\langle \tau \rangle^2} \tag{1}$$

where τ is the carrier collision time.

When τ is assumed to be energy independent, $\langle \tau^2 \rangle = \langle \tau \rangle^2$ and $r_H = 1$ [[**?**]]. This approximation is commonly made for semiconductor materials during experimental Hall measurements, allowing the measured Hall mobility, μ_H, to be considered equal to the conductivity mobility of the material.

Unlike these materials, graphene exhibits a linear dispersion relation around the Dirac point. This leads to a significant energy dependence of the carrier scattering rate. Based on the Boltzmann Transport Equation and considering carrier scattering rate due to acoustic and optical phonons, a theoretical study shows that r_H in graphene strongly depends on doping and temperature. This factor can reach values as high as $r_H \approx 4$ at low doping concentration below $300K$ [[**?**]]. Nevertheless, to the best of our knowledge, such high scattering factors have not been reported experimentally. This is probably because they are expected to occur in regions where electrons and holes coexist due to thermal activation and/or inhomogeneities in the device.
